# DiaNet v2 deep learning based method for diabetes diagnosis using retinal images

**DOI:** 10.1038/s41598-023-49677-y

**Published:** 2024-01-18

**Authors:** Hamada R. H. Al-Absi, Anant Pai, Usman Naeem, Fatma Kassem Mohamed, Saket Arya, Rami Abu Sbeit, Mohammed Bashir, Maha Mohammed El Shafei, Nady El Hajj, Tanvir Alam

**Affiliations:** 1 College of Science and Engineering, Hamad Bin Khalifa University, Doha, Qatar; 2 Ophthalmology Section, Department of Surgery, Hamad Medical Corporation, Doha, Qatar; 3 Endocrine Section, Department of Medicine, Hamad Medical Corporation, Doha, Qatar; 4 Qatar Metabolic Institute, Hamad Medical Corporation, Doha, Qatar; 5 College of Health and Life Sciences, Hamad Bin Khalifa University, Doha, Qatar

**Keywords:** Diabetes, Computational science

## Abstract

Diabetes mellitus (DM) is a prevalent chronic metabolic disorder linked to increased morbidity and mortality. With a significant portion of cases remaining undiagnosed, particularly in the Middle East North Africa (MENA) region, more accurate and accessible diagnostic methods are essential. Current diagnostic tests like fasting plasma glucose (FPG), oral glucose tolerance tests (OGTT), random plasma glucose (RPG), and hemoglobin A1c (HbA1c) have limitations, leading to misclassifications and discomfort for patients. The aim of this study is to enhance diabetes diagnosis accuracy by developing an improved predictive model using retinal images from the Qatari population, addressing the limitations of current diagnostic methods. This study explores an alternative approach involving retinal images, building upon the DiaNet model, the first deep learning model for diabetes detection based solely on retinal images. The newly proposed DiaNet v2 model is developed using a large dataset from Qatar Biobank (QBB) and Hamad Medical Corporation (HMC) covering wide range of pathologies in the the retinal images. Utilizing the most extensive collection of retinal images from the 5545 participants (2540 diabetic patients and 3005 control), DiaNet v2 is developed for diabetes diagnosis. DiaNet v2 achieves an impressive accuracy of over 92%, 93% sensitivity, and 91% specificity in distinguishing diabetic patients from the control group. Given the high prevalence of diabetes and the limitations of existing diagnostic methods in clinical setup, this study proposes an innovative solution. By leveraging a comprehensive retinal image dataset and applying advanced deep learning techniques, DiaNet v2 demonstrates a remarkable accuracy in diabetes diagnosis. This approach has the potential to revolutionize diabetes detection, providing a more accessible, non-invasive and accurate method for early intervention and treatment planning, particularly in regions with high diabetes rates like MENA.

## Introduction

Diabetes mellitus (DM) is a chronic metabolic disorder characterized by Hyperglycaemia and is associated with increased long term morbidity and mortality^1,2^. According to the International Diabetes Federation (IDF), there were 537 million people affected by diabetes worldwide in 2021 and this number is expected to be increased to more than 600 million by 2030. In Middle East and North Africa (MENA), there were 73 million people affected by diabetes in 2021 with an expectation of 87% increase in the cases to reach 136 million by 2045^3^. The two main type of DM are type 1 DM (DM-1) and type 2 DM ( DM-2), the latter accounts for almost 90% of the cases^4^. Early detection of diabetes has a big impact on treatment and prevention of further complications, however, a report by the IDF has indicated that about 50% of people affected with diabetes in 2021 were undiagnosed and unaware^5^.

To diagnose diabetes, healthcare professionals have been using tests such as fasting plasma glucose (FPG), oral glucose tolerance tests (OGTT), random plasma glucose (RPG), and hemoglobin A1c (HbA1c)^6^. Although these tests are widely used, they have some limitations. For example, FPG has been reported to have lower sensitivity for diabetes detection^7^. In fact, a report by the World Health Organization and IDF^8^ stated that 30% of undiagnosed diabetes were missed using FPG. Furthermore, it is mandatory to be fasting for a person to take this exam for at least 8 h, and this might be inconvenient for some people. Due to its poor reproducibility, it is recommended to repeat FPG within 3 months time^9^. OGTT is lobor-intensive and time-consuming and it needs to be administered under specific conditions for the results to be accurate, including specific diet prior to the test and to ensure that a 2-h sample is collected within 5 min of 120 min^8^. Moreover, 12% of people who are tested with OGTT are misclassified as either diabetic or suffer from impaired glucose tolerance (IGT)^10^. For the RPG, although this test could be taken at any time without conditions such as fasting, the National Institute of Diabetes and Digestive and Kidney Diseases^11^ has stated that it has greater variability within the same patient, affected by changes in lifestyle and dietary and is less sensitive in measuring diabetes. The HbA1c is currently the gold standard for diabetes detection as it reports the average blood glucose. According to the American Diabetes Association (ADA), HbA1c has lower sensitivity at a designated cut point, it may not be available in certain regions of the developing world, it is costly, its measurement can be interfered by hemoglobin variants^12^. HbA1c results might be impacted by any form of anaemia or hemoglobinopathy and a poor correlation between HBA1c and glucose parameters is very common, which can cause confusion with the diagnosis^13^.

Considering the above backdrop, exploring alternative affordable and with easy access methods to diagnose diabetes (especially in middle- and low-income nations) with high accuracy is needed. There exist multiple studies that have used alternative ways to diagnose diabetes using electrocardiography (ECG)^14^, retinal images^15,16^ and breath test^17^; other methods that have also been explored include using saliva, sweat and tears^18^. Previously, we developed the very first deep learning model DiaNet for diabetes detection using retinal images only^15^. The proposed model, based on retinal images of 500 participants from QBB, achieved an accuracy of 84% in distinguishing diabetic from healthy individuals.

Recently retinal image has gained a lot of attention in the scientific community for the detection of cardiovascular disease^19^, diabetic retinopathy (DR)^20,21^ and other diseases^22^. In this article, we incorporated the largest cohort from Qatar Biobank (QBB) and Hamad Medical Corporation (HMC) to improve the prediction model for diabetes diagnosis. The contribution of this work can be summarized as follows:

1. We have used the largest collection of retinal images from more than 5000 patients/participants to build a diabetes diagnosis model DiaNet v2 based on retinal images only. The proposed VGG-11-based DiaNet v2 model outperformed the previous model and achieved over 92% accuracy in distinguishing diabetic patients from the control group.
2. We validated the proposed model retrospectively using a retinal image dataset from HMC, the largest healthcare provider in Qatar, and it shows that retinal images can be considered as an excellent source for the diagnosis of diabetes.

## Results

In this section, we present the results obtained on multiple experiments that we conducted using multiple DL models on the retinal images for diabetes classification. Given the change and expansion of our image dataset, there is a possibility that the deep learning architecture we used in DiaNet v1 is not optimal for these new images. As a result, we tested a transfer learning approach with five common deep learning architectures in order to find the one that performs the best on our new dataset.

### Performance of the proposed models for diabetes diagnosis based on retinal images

**Figure 1:**
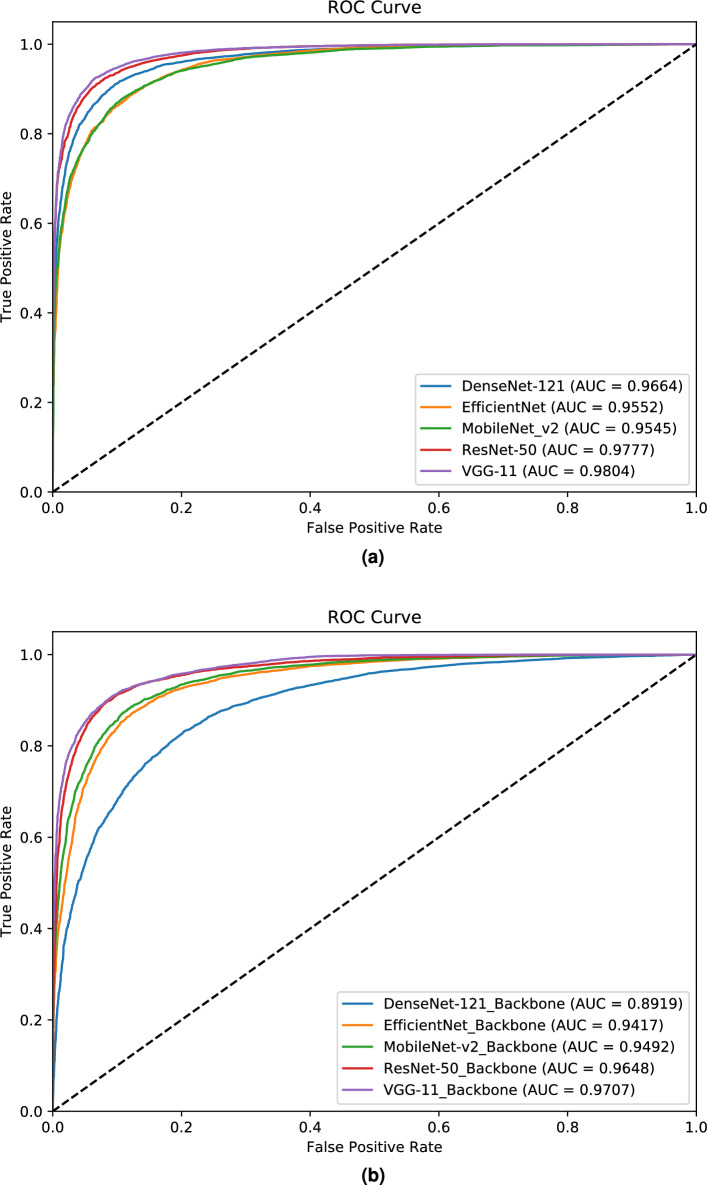
ROC plot for the modified models (**a**) and backbone models (**b**).

Table 1 presents the average performance metrics obtained from a 5-fold cross-validation analysis for diabetes prediction using five deep learning models: DenseNet-121, ResNet-50, EfficientNet, VGG-11, and MobileNe_v2. The table shows the results from backbone model as well as modified network that we propose as part of DiaNet v2. The performance metrics evaluated for each model include accuracy, sensitivity, specificity, precision, F1-score and Matthew’s correlation coefficient (MCC). These metrics provide insights into the models’ effectiveness in distinguishing between individuals with diabetes and those without diabetes. The result also shows the p value to measure statistical significance for each model’s performance metric before and after the modification of network. The results indicate that all the modified models achieved higher level of accuracy, ranging from 88.12 to 92.63%, when compared to the backbone models which achieved accuracy ranging from 81.08 to 90.59%. This suggests that superiority of the modified network compared to the backbone models in classifying a larger portion of the diabetes and control samples. The modified VGG-11 model achieved the highest accuracy with 92.63%. In terms of sensitivity, which measures the ability to correctly identify individuals with diabetes, the modified network achieved values between 85.56 and 93.93% compared to values between 81.81 and 94.46% for the backbone models. On the other hand, specificity, which measures the ability to correctly identify individuals without diabetes, ranged from 90.69 to 91.31% for the modified network compared to values ranging from 80.36 to 90.53% for the backbone models. Summarily, these results demonstrate that the modified network performed well and generally better than the backbone models in both identifying individuals with diabetes and without the condition. The F1-score, which combines precision and sensitivity, ranged from 87.76 to 92.81% for the modified models compared to values ranged from 81.23 to 90.58% for the backbone models. This metric provides a balanced assessment of the models’ performance, considering both the ability to correctly identify positive samples and minimize false positives. The modified VGG-11 achieved the highest F1-score of 92.81%. The Matthews correlation coefficient (MCC), which takes into account true and false positives and negatives, ranged from 76.37 to 85.32% for the modified models compared to values from 62.18 to 81.32% for the backbone models. A higher MCC value indicates a better overall performance of the model. With regard to the statistical significance testing, there was a statistical difference for each pair of models for performance metrics with p values (< 0.001). Figure 1 shows the area under the curve (AUC) of receiver operating characteristics (ROC) curve for the models before and after the modification. The modified models’ AUC values ranged from 95.52 to 98.04%, where the backbone models’ AUC ranged from 89.19 to 97.07%. A higher AUC value suggests a better discriminative ability of the model. The modified VGG-11 achieved the highest AUC with 98.04%.

**Table 1:** Ablation study showing performance of different DL models before and after modification of network backbone.

Model	Result type	Accuracy	Sensitivity	Specificity	Precision	F1-score	MCC
DenseNet-121	Modified (DiaNet v2)	0.9048	0.8925	0.9172	0.9134	0.9021	0.8108
	Backbone	0.8108	0.8181	0.8036	0.8068	0.8123	0.6218
	p value	5.669E−154	5.888E−183	9.462E−147	7.443E−136	7.571E−173	2.677E−136
ResNet-50	Modified (DiaNet v2)	0.9213	0.9184	0.9241	0.9231	0.9205	0.843
	Backbone	0.9059	0.9065	0.9053	0.9068	0.9058	0.8132
	p value	1.666E−144	7.275E−139	2.175E−113	7.199E−117	3.827E−142	1.332E−144
EfficientNet	Modified (DiaNet v2)	0.8812	0.8556	0.9069	0.9011	0.8776	0.7637
	Backbone	0.874	0.8735	0.8746	0.8765	0.874	0.7497
	p value	3.625E−153	1.764E−106	7.987E−149	1.053E−160	1.027E−155	2.261E−152
VGG-11	Modified (DiaNet v2)	**0.9263**	0.9393	0.9132	0.9176	**0.9281**	**0.8532**
	Backbone	0.8914	0.9446	0.838	0.8781	0.9041	0.7978
	p value	2.375E−232	1.574E−235	9.616E−17	9.175E−29	2.505E−206	3.565E−187
MobileNet_v2	Modified (DiaNet v2)	0.8842	0.8918	0.8765	0.8784	0.8849	0.7687
	Backbone Network	0.8822	0.8814	0.8829	0.883	0.8821	0.7646
	p value	4.069E−159	6.224E−158	4.146E−74	8.861E−80	7.673E−150	9.739E−160

Highest values are in [bold].

### Performance of the models based on gender-stratified samples

**Figure 2:**
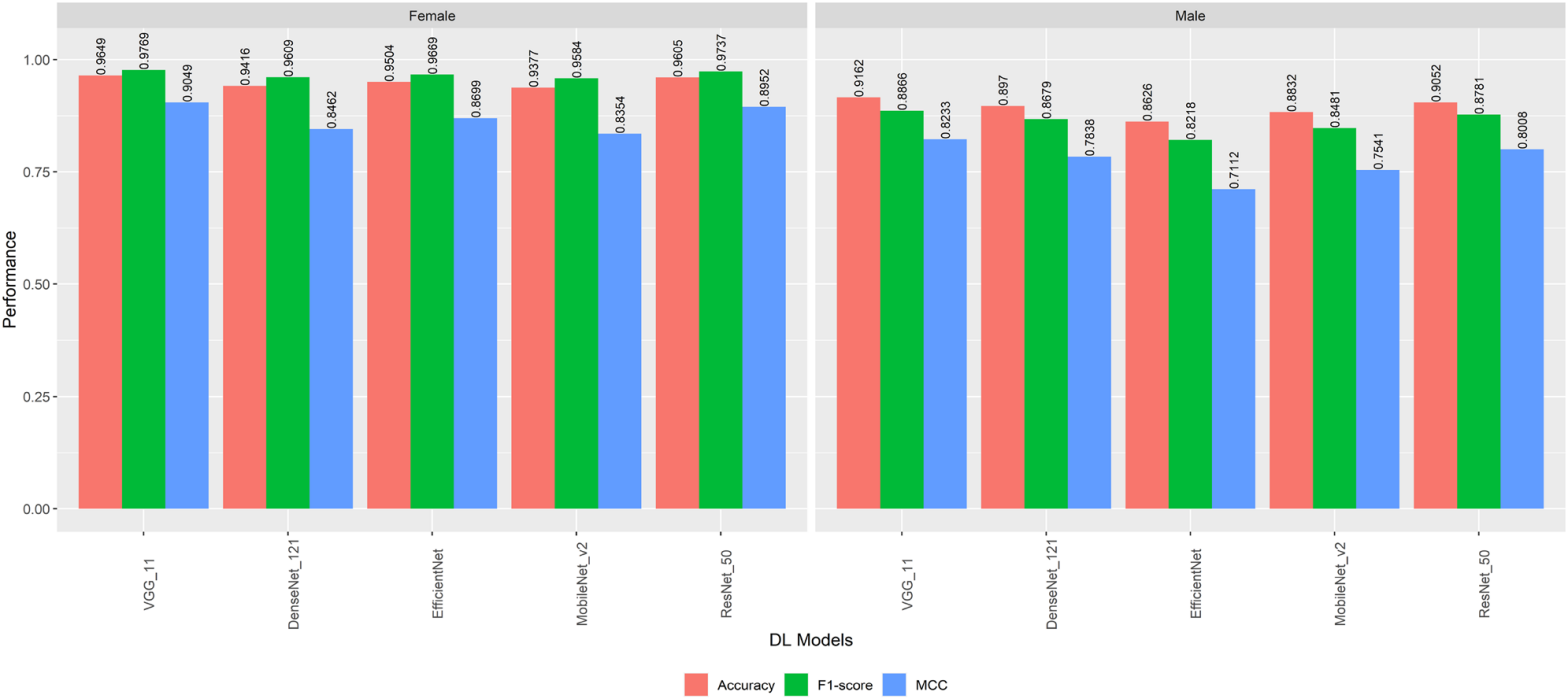
Performance of different deep learning models based on gender-stratified groups.

We experimented with the DL models’ performance considering age- and gender-stratification samples. VGG-11 model achieved the highest accuracy, F1 Score and MCC with 96.49%, 97.69% and 90.49% respectively on female participants (Fig. 2). VGG-11 achieved the highest accuracy, F1 Score and MCC as well on male participants with 91.62%, 88.66%, and 82.33%, respectively. Generally, all models achieved better performance on female participants compared to male participants, considering the gender-stratified sample.

### Performance of the models based on age-stratified samples

**Figure 3:**
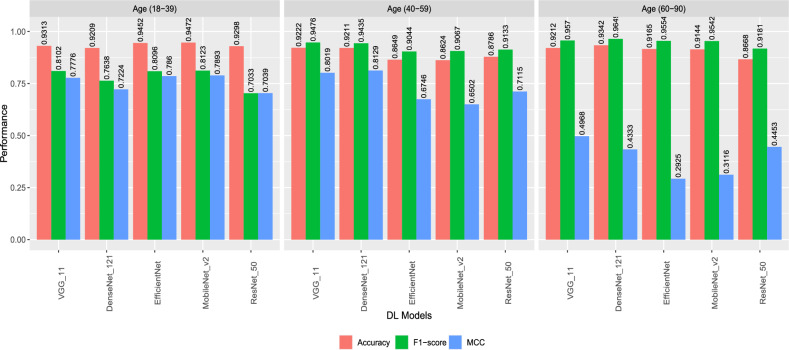
Performance of different deep learning models based on age-stratified groups.

Considering the age-stratified sample, VGG-11 performed better than other models in terms of accuracy in different age groups, with 93.13% in the (18–39) age group, 92.22% in the (40–59) age group, and 92.12% in the (60–90) age group (Fig. 3). In terms of F1-Score, the DL models achieved consistent values for the (40–59) age group (between 90.67% with MobileNet_v2 and 94.76% with VGG-11) (Fig. 3). The performance for the (60–90) age group achieved the lowest MCC values (i.e below 50%) compared to other age groups. This is due to the few control cases in this group, where there were only 116 control compared to 1812 diabetes cases (Fig. 3). Based on these results, we may conclude that the DL models were able to detect diabetes in the (18–39) and (40–59) age groups compared to the (60–90) age group (Fig. 3). Furthermore, a statistical significance testing across all age groups shows that most of the results were statistically significant as shown in Table 2.

**Table 2:** Performance of models on age-stratified dataset.

Age group	Metric	DenseNet_121	ResNet_50	EfficientNet	VGG_11	MobileNet_v2
Age (18–39)	Accuracy	0.9209	0.9298	0.9452	0.9313	0.9472
Age (40–59)	Accuracy	0.9211	0.8786	0.8649	0.9222	0.8624
Age (60–90)	Accuracy	0.9342	0.8668	0.9165	0.9212	0.9144
Across age groups p value for Accuracy		6.96E−230	0.386	1.31E−17	1.73E−69	1.418E−89
Age (18–39)	F1-score	0.7638	0.7033	0.8096	0.8102	0.8123
Age (40–59)	F1-score	0.9435	0.9133	0.9044	0.9476	0.9067
Age (60–90)	F1-score	0.9649	0.9181	0.9554	0.957	0.9542
Across age groups p value for F1-score		<0.001	1.27E−30	5.76E−99	2.49E−13	1.260E−135
Age (18–39)	MCC	0.7224	0.7039	0.786	0.7776	0.7893
Age (40–59)	MCC	0.8129	0.7115	0.6746	0.8019	0.6502
Age (60–90)	MCC	0.4333	0.4453	0.2925	0.4968	0.3116
Across age groups p value for MCC		3.65E−234	0.0014	0.0065	4.37E−59	1.173E−112

### Class activation map highlighting the region of interest for DiaNet v2

**Figure 4:**
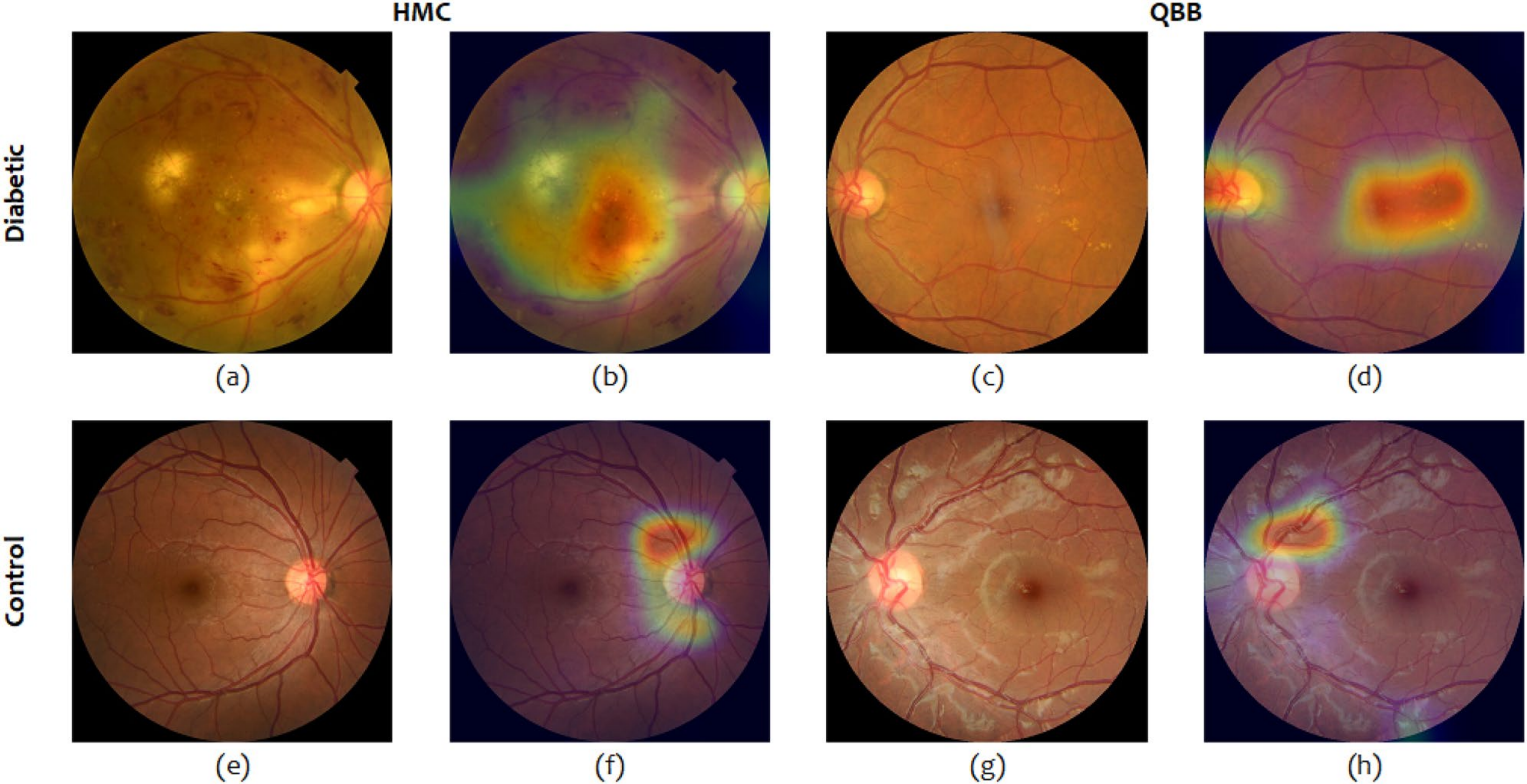
Retinal images with overlaid heatmap. Images a the top (**a**) to (**d**) show examples of diabetic images. Images at the bottom (**e**) to (**h**) show examples of control (non-diabetic) image. Images on the left are the original input images while those on the right are the corresponding class activation map (CAM).

The provided visual representation, displayed in Fig. 4, illustrates retinal images and superimposed heatmaps derived from both the diabetes and control cohorts. The heatmaps, color-coded for distinct degrees of influence on predictions across the images, highlight areas of significance. The images selected for inclusion in Fig. 4 indicate strong predictions (probability exceeding 0.80) from the DiaNet v2 model. Within Fig. 4, the upper and lower rows showcase retinal images from the diabetes and control groups, respectively. Notably, across all these images, the superimposed heatmaps primarily concentrate on critical areas such as the optic disc, macula, and intermediate zone–locations prone to exhibiting DR characteristics. Of note, Fig. 4b,d reveal extensive microaneurysms, minute bulges originating from smaller vessel walls, representing the earliest clinically detectable indications of DR. Additionally, all three images depict intra-retinal hemorrhages and exudation, common features in individuals affected by DR.

Furthermore, Fig. 4b presents tortuous retinal blood vessels marked by arteriovenous nicking and venous dilation, indicators associated with systemic conditions like hypertension, diabetes, and ischemic heart disease. This strengthens our assertion that our model effectively identifies general retinal diabetes-related attributes. These signs are in line with medical findings indicating that diabetic eye can suffer from signs like small blood vessel damage (microaneurysms), swelling of the retina (edema), white-yellow deposits (exudates), bleeding in the retina (hemorrhages), cloudy spots (cottonwool spots), and areas with reduced blood flow^23–25^. In contrast, the control group images (Fig. 4e–h) lack these distinctive features in the highlighted regions, indicating the absence of diabetes-related symptoms in these retinas.

### Comparison against other existing results

In 2021, we introduced DiaNet, an initial deep learning model that achieved an AUC of 0.84, according to our publication^15^. The development of this model involved analyzing data from 500 participants in the QBB dataset. Subsequently, we expanded our research by including a significantly larger sample size of 5000 participants from the QBB dataset, a tenfold increase. Additionally, we incorporated data from diabetic patients with various pathological characteristics at the HMC Ophthalmology clinic. As a result, we confidently assert that the dataset used for our study and the upgraded DiaNet version 2 model described in this current paper are notably more robust and accurate. Notably, DiaNet v2 achieves an impressive AUC of 0.98, surpassing the performance of the initial DiaNet model. In 2022, Yun et al. undertook a project involving 12,185 participants from the UK Biobank to develop a deep learning model for diabetes diagnosis, as documented in^26^. Their chosen architecture was a ResNet-18-based model, which achieved an AUC of 0.703^26^. It’s important to highlight that due to limitations in data access, we were unable to utilize retinal images from the UK Biobank dataset to evaluate our model. In the present study, we overcame this limitation by utilizing an extensive collection of retinal images from a substantial number of patients in Qatar. Our dataset consists of 5545 participants covering both the diabetic and the control group. With this enriched dataset, we constructed the DiaNet v2 model, which demonstrated exceptional performance with an AUC of 0.98. This unequivocally demonstrates the superior predictive capability of our model within the Qatari population dataset.

## Discussion

The global burden of diabetes has been escalating, with alarming projections of rising prevalence rates. Particularly concerning is the trend observed in the Middle East and North Africa (MENA) region. The significance of early diabetes detection cannot be overstated, as it allows for timely intervention to prevent further complications. However, the challenges associated with current diagnostic methods in clinical setup, such as FPG, OGTT, RP, and HbA1c, have led to significant limitations in their effectiveness^6^. To address these limitations and explore alternative methods, we turned to retinal images as a potential avenue for diabetes diagnosis. The utility of retinal images for detecting various diseases, including diabetes, has garnered substantial attention in recent years. Therefore, Building on our prior work^15^, where we developed DiaNet, the first deep learning model for diabetes detection using retinal images, we expanded our approach in this study to propose DiaNet v2.

In DiaNet v2, we leveraged the largest collection of retinal images from the Qatari population to develop an improved model, achieving over 92% accuracy in distinguishing diabetic patients from the control group. This is a notable advancement over our previous model, signifying the value of increasing the dataset size and refining the model architecture. We also validated the performance of DiaNet v2 using a dataset from the Hamad Medical Corporation (HMC), affirming the robustness of retinal images as an excellent source for diabetes diagnosis. The dataset utilized in this research possesses a unique attribute, as it encompasses retinal images sourced from both a biobank and a hospital setting. Within the context of the QBB, the retinal images lack annotations provided by ophthalmologists pertaining to any preexisting pathologies. Consequently, we are devoid of valuable information regarding prior ocular pathologies concerning the QBB participants. In order to address this inherent limitation of the QBB dataset, we have integrated a dataset from the HMC. This particular dataset has been curated and annotated by ophthalmologists affiliated with the HMC, resulting in an enhancement of dataset quality. The images within this dataset exhibit a range of existing pathologies linked to diabetes, as well as other pathologies evident in retinal images that are unrelated to diabetes. For example, in Fig. 6a, the image belongs to the diabetes group and has vitreous hemorrhage, which is a consequence of being diabetic^27^. Figure 6b is another example of a diabetic eye affected by microaneurysm which is an early sign of DR^28^. Figure 6c shows a diabetic eye with mild nonproliferative diabetic retinopathy (NPDR), which is an early stage of DR^29^ . Figure 6d–f shows examples of non-diabetic eyes that have glaucoma in (d), trauma caused by laser in (e), and retinal detachment in (f). All these pathologies has helped in building a model that is capable of distinguishing diabetic from non-diabetic patients based on retinal images only.

Moreover, the gender-stratified version of DiaNet v2 revealed interesting trends in model performance. Across all models, higher accuracy, F1-scores, and MCC values were consistently observed in female participants. This gender disparity in model performance warrants further investigation and may be attributed to physiological and biological differences between genders. Similarly, the age-stratified analysis demonstrated that VGG-11 exhibited superior accuracy across age groups, with the highest accuracy achieved in the (18–39) and (40–59) age groups. However, the model’s performance in the (60–90) age group was hindered by a smaller control group size, indicating the importance of balanced datasets for accurate evaluation. The Class Activation Map (CAM) analysis unveiled critical regions within retinal images that significantly influence the DiaNet v2 model’s predictions. These regions primarily encompass the optic disc, macula, and intermediate zone-areas vulnerable to DR manifestations. This CAM analysis revealed evidence of systemic conditions like hypertension, diabetes, and ischemic heart disease, reinforcing the notion that retinal images offer valuable insights into general retinal attributes related to diabetes.

This study utilized a dataset gathered from residents of Qatar, predominantly comprising individuals of Middle Eastern descent. As a result, the findings might lack generalizability to other populations at the similar level of Middle Eastern descent. Nevertheless, the study underscores the considerable promise of early diabetes detection using retinal images considering its non-invasive, inexpensive, and fast screening nature. One more limitation in our research is the absence of a comparison between the suggested AI model’s performance and human-level intelligence in diabetes screening solely based on retinal images. To accomplish this, we will need to form a separate group of Ophthalmologists with varying expertise levels from different medical facilities. Subsequently, a comparison between the AI model and human performance would be conducted. This aspect will be part of our upcoming steps in the near future.

In conclusion, our study contributes significantly to the field of diabetes diagnosis by demonstrating the potential of deep learning models in utilizing retinal images as a reliable and non-invasive tool. The high accuracy, sensitivity, and specificity achieved across various models highlight the promise of this approach. Gender- and age-stratified analyses shed light on performance disparities and demographic influences, prompting further research into these factors. With the potential to revolutionize diabetes diagnosis, retinal image-based methods offer a pathway to accessible and effective early detection, especially in regions with limited resources. Future studies should focus on addressing biases, exploring multi-modal approaches, and conducting prospective clinical validations to establish the real-world utility of these models.

## Methods

### Dataset collection

**Figure 5:**
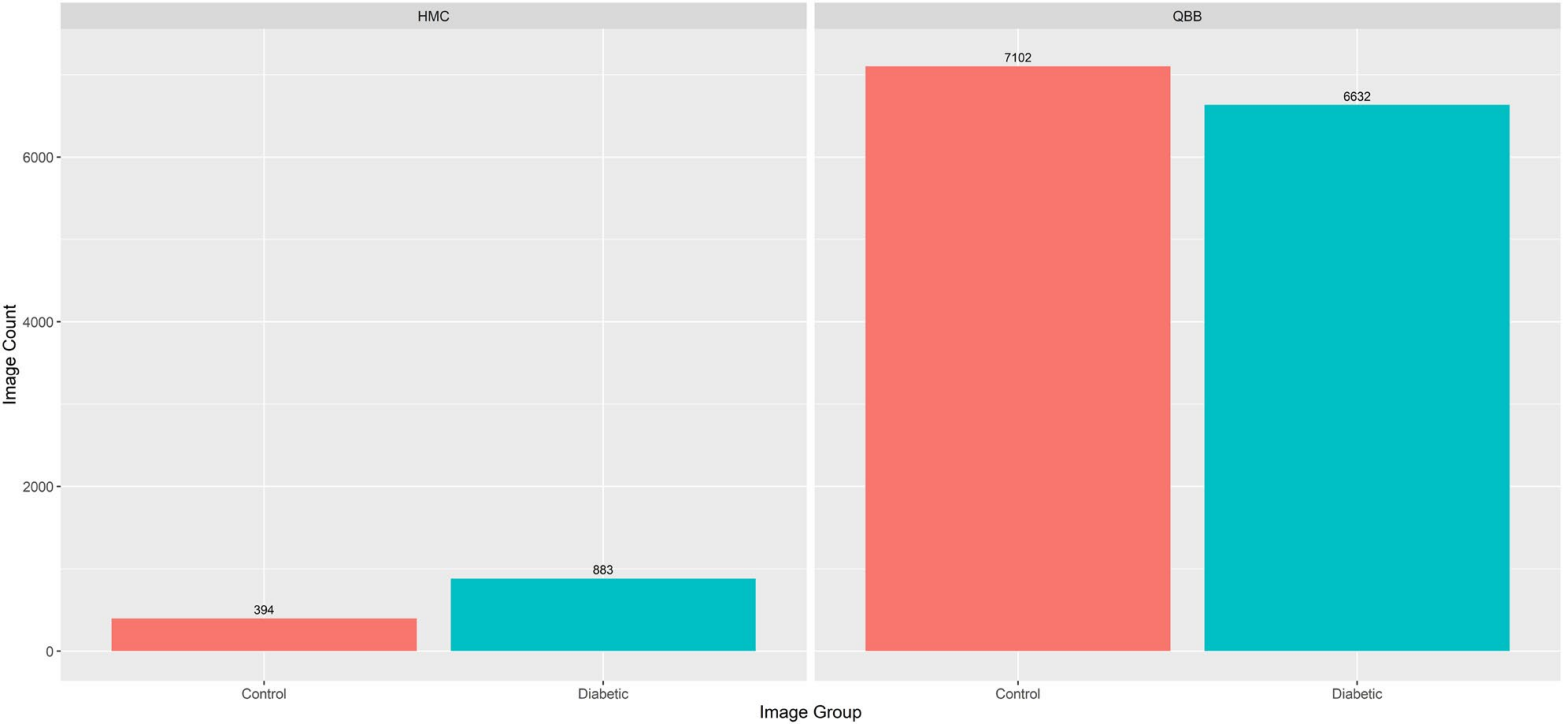
Summary statistics of images used in the study from HMC and QBB.

For this study, a total number of 15,011 images were collected, where 7515 images were for diabetic and 7496 images for control (Fig. 5). These images were collected from two sources: (1) Hamad Medical Corporation and (2) Qatar Biobank. Further details about both sources are given below.

**Figure 6:**
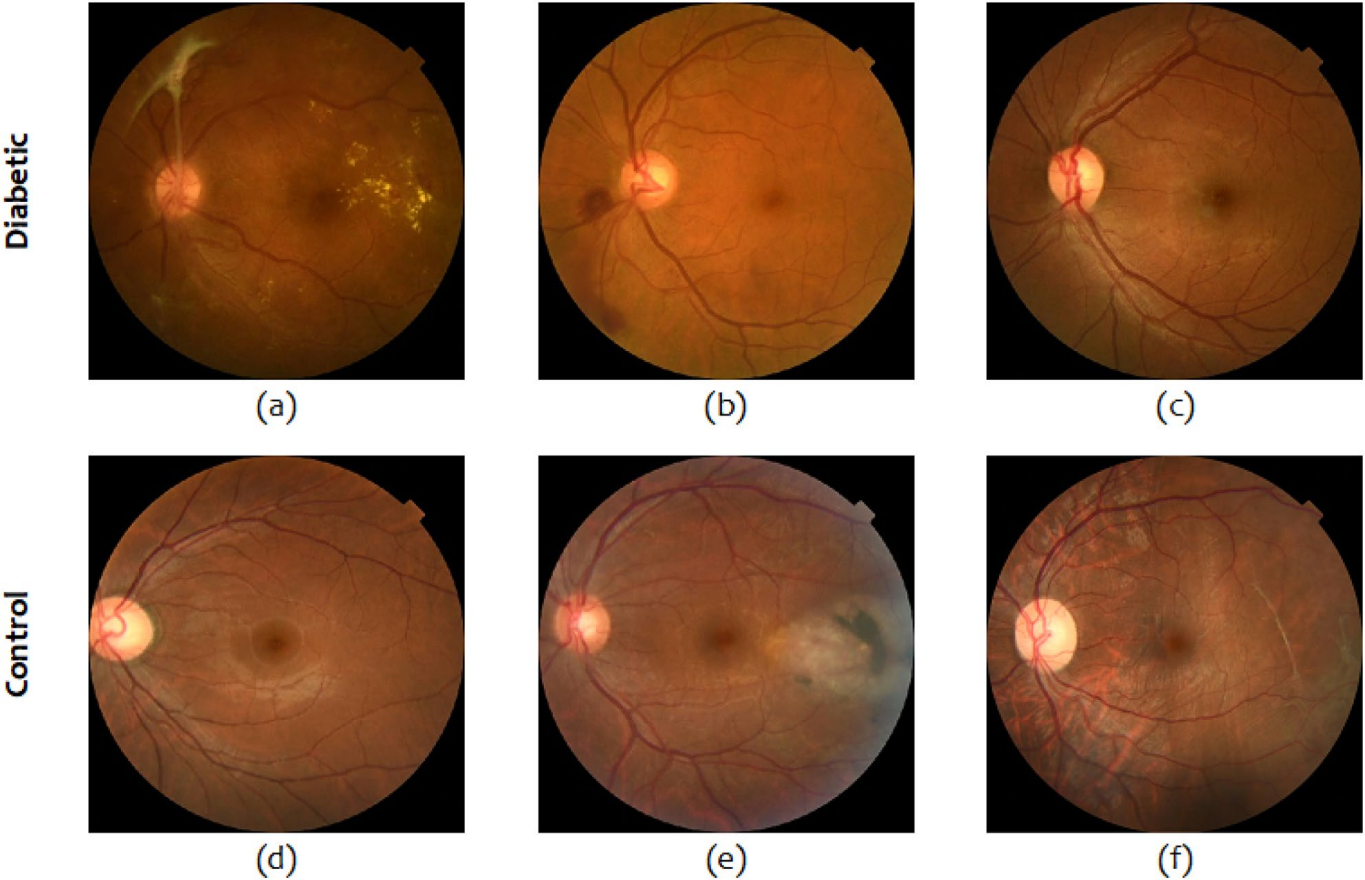
Sample of retinal images used in this study. Presented pathologies include: hemorrhage (**a**), microaneurysm (**b**), mild NPDR (**c**), glaucoma (**d**), laser caused trauma (**e**) and retinal detachment (**f**).

#### Retinal images from Hamad Medical Corporation

We collected retinal images from the ophthalmology department at Hamad Medical Corporation (HMC). The dataset contains retinal images for 641 HMC diabetic patients (HbA1C $\ge$ 6.5) as well as non-diabetic patients who have visited HMC between January 1st, 2012, and December 31st, 2021. Retina images of patients with and without diabetes were collected with some biographical data such as age, country, and HbA1c. Among the collected data, there were 442 diabetic patients, covering a total of 883 retinal images, and 199 non-diabetic patients, covering a total of 396 retinal images. Only six images were removed from the dataset due to bad quality after manual inspection or were unusable (i.e., wide-angle view images). Figure 6 shows example of images collected from HMC.

The image data of diabetic patients contained retinal pathologies related to diabetes, such as non-proliferative diabetic retinopathy, proliferative diabetic retinopathy (PDR), PDR treated by laser therapy, etc. This group also consisted of images with no visible pathological signs. The image data of the non-diabetic group consisted of images with no visible pathological signs as well as fundus images of different non-diabetic pathologies. These different non-diabetic pathologies included macular scar, choroidal neovascular membranes, retinal detachment, age-related macular degeneration, choroiditis, and central serous retinopathy. The collection of the images for this study was approved by HMC’s Institutional Review Board (IRB) (approval number: MRC-03-22-279). Due to the retrospective nature of the study, informed consent was waived.

#### Retinal images from Qatar Biobank

From Qatar BioBank (QBB), we collected a dataset of 4905 participants covering diabetes (with HbA1C $\ge$ 6.5) and a control group. We had, in total, 2099 diabetic participants and 2806 control participants in this QBB cohort. Each participant has at least one retinal image and up to four images. The total number of images was more than 18,000 images initially; however, after a quality check, we removed around 2000 images due to bad quality. The images were collected under the regulation of the Ministry of Public Health, Qatar. The Institutional Review Board of Qatar Biobank, Qatar, approved this study, and only a de-identified dataset was collected from QBB. Details of data collection can be found in^30,31^. Then, we combined datasets from both HMC and QBB covering a total of 15,011 retinal images. The dataset used for the analysis consisted of 7496 retinal images from the control group and 7515 retinal images from the diabetes group.

#### Retinal image pre-processing

Many of the images were large and had an extended black background on the sides, so these were cropped, creating squared images with the size of 540 $\times$ 540. All retinal images were then processed by subtracting the local mean from 4 $\times$ 4 neighboring pixels. This is based on the method that was proposed by Graham^32^. To increase the robustness of our model, Random flipping horizontally and vertically well as random brightness and contrast, were applied.

### DiaNet v2: the proposed deep learning model architecture

**Figure 7:**
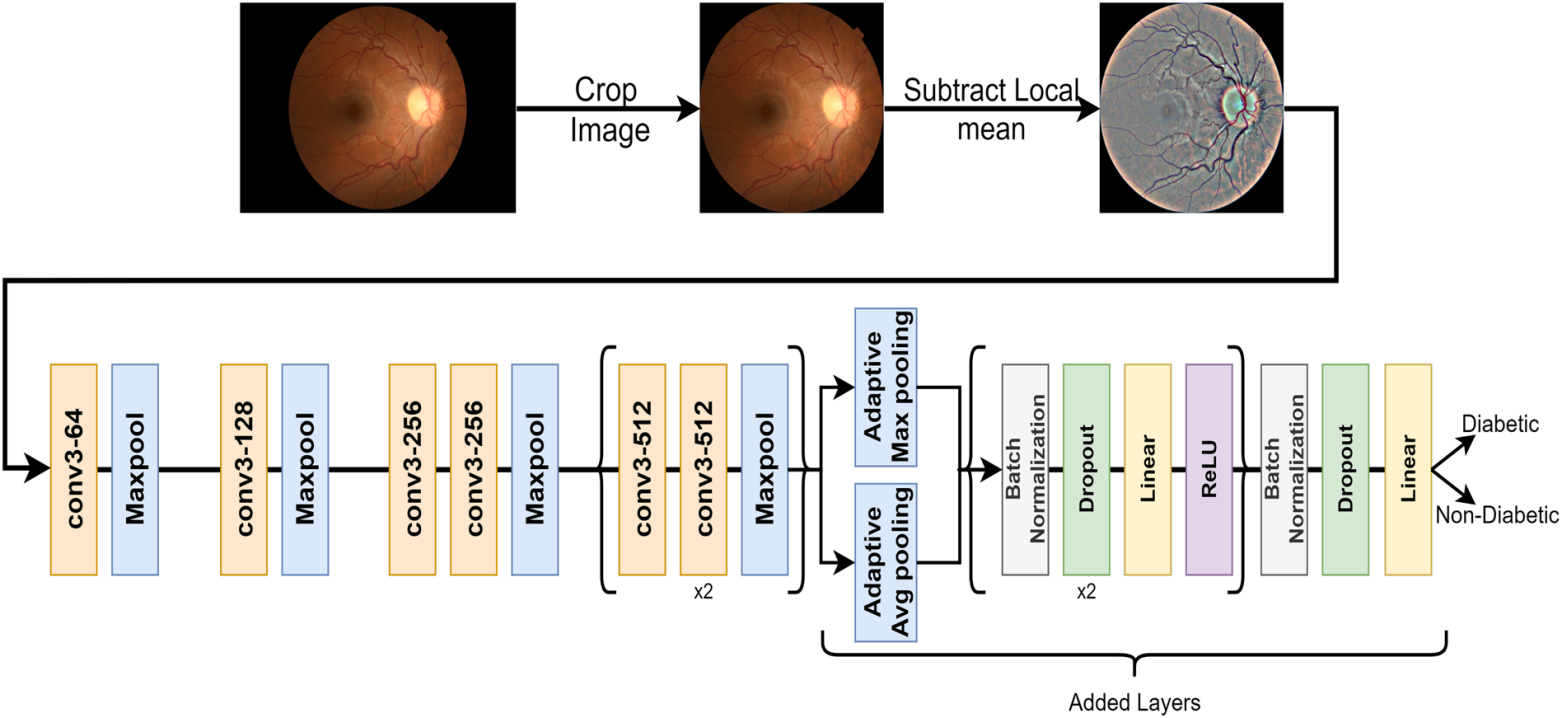
A diagram showing the overall flow of the deep learning model to diagnose diabetes. Up: all images are cropped and then processed by subtracting the local mean from a 4 $\times$ 4 neighboring pixels. Down: the architecture of the deep learning model with VGG-11 as the backbone of the model.

We developed deep learning models based on a transfer learning approach with five different networks, i.e., DenseNet-121, ResNet-50, EfficientNet, VGG-11, and MobileNet_v2. We achieved the best results with VGG-11 network^33^, which was trained with ImageNet and has an output of 1000 neurons in its final layer. VGG-11 is known for its simplicity and effectiveness in image classification; it uses small convolution filters of 3 $\times$ 3, which capture local patterns of an image and its details^33^. Since our aim here is to distinguish diabetic from non-diabetic images, our network’s output should have only two neurons in the output layer. To enhance the outcome of this network, replaced the fully connected layers with layers as follows: a global average pooling layer and a global max pooling layer were added, and their output was concatenated and then followed by two sequences of batch normalization, dropout, linear, and ReLU layers. And finally, a dropout layer was followed by a linear layer with two neurons as the output. Figure 7 presents a simple block diagram of the proposed VGG-11 architecture^33^. We utilized the Adam optimizer with a learning rate of 1e−4 to gradually fine-tune the model’s parameters during training, promoting effective pattern learning. We used a batch size of 64 for 100 epochs with early stopping to prevent overfitting. To conduct all experiments, we used a workstation with 12th Gen Intel(R) Core (TM) i7-12700KF, with 128 GB RAM and GeForce RTX 3090 GPU. We used Python version 3.10.4 with PyTorch^34^ version 1.11.0 to implement the deep learning model. Unlike DiaNet v1, DiaNet v2 was trained on only the combined dataset from HMC and QBB. EyePACS^35^ dataset was used in DiaNet v1 to finetune the network before training it with the small QBB dataset that was used in the previous publication^15^.

## Ethical approval

The study was conducted in accordance with the Declaration of Helsinki, and approved by the Institutional Review Board of Hamad Medical Corporation and Qatar Biobank. Due to the retrospective nature of the study, informed consent was waived from HMC.

## Data Availability

The datasets generated and/or analysed during the current study are not publicly available due non-disclosure agreement. But they can be accessed through application to the Qatar Biobank and Hamad Medical Corporation through an established ISO-certified process by submitting a request online, subject to institutional review board approval by the Qatar Biobank and Hamad Medical Corporation. Users can contact the corresponding author or send email to qbbresearch@qf.org.qa to raise request for accessing dataset.
